# Preventing HIV among adolescents with oral PrEP: observations and challenges in the United States and South Africa

**DOI:** 10.7448/IAS.19.7.21107

**Published:** 2016-10-18

**Authors:** Sybil Hosek, Connie Celum, Craig M Wilson, Bill Kapogiannis, Sinead Delany-Moretlwe, Linda-Gail Bekker

**Affiliations:** 1 Department of Psychiatry, Stroger Hospital of Cook County, Chicago, IL, USA; 2 Department of Global Health, University of Washington, Seattle, WA, USA; 3 Department of Epidemiology, University of Alabama at Birmingham, Birmingham, AL, USA; 4 Maternal and Pediatric Infectious Diseases Branch, Eunice Kennedy Shriver National Institutes of Child Health and Human Development, Washington, DC, USA; 5 RHI, University of Witwatersrand, Johannesburg, South Africa; 6 The Desmond Tutu HIV Centre, University of Cape Town, Cape Town, South Africa

**Keywords:** PrEP, adolescents, HIV/AIDS, implementation

## Abstract

**Introduction:**

Adolescents and young adults aged <25 are a key population in the HIV epidemic, with very high HIV incidence rates in many geographic settings and a large number who have limited access to prevention services. Thus, any biomedical HIV prevention approach should prepare licensure and implementation strategies for young populations. Oral pre-exposure prophylaxis (PrEP) is the first antiretroviral-based prevention intervention with proven efficacy across many settings and populations, and regulatory and policy approvals at global and national levels are occurring rapidly. We discuss available data from studies in the United States and South Africa on the use of oral PrEP for HIV prevention in adolescent minors, along with some of the implementation challenges.

**Discussion:**

Ongoing studies in the United States and South Africa among youth under the age of 18 should provide the safety data needed by the end of 2016 to contribute to licensure of Truvada as daily PrEP in adolescents. The challenges of completing these studies as well as foreseeable broader challenges highlighted by this work are presented. Adherence to daily PrEP is a greater challenge for younger populations, and poor adherence was associated with decreased efficacy in all PrEP trials. Individual-level barriers include limited familiarity with antiretroviral-based prevention, stigma, product storage, and social support. Structural challenges include healthcare financing for PrEP, clinician acceptability and comfort with PrEP delivery, and the limited youth-friendly health services available. These challenges are discussed in the context of the work done to date in the United States and South Africa, but will likely be magnified in the setting of limited resources in many other countries that are heavily impacted by HIV.

**Conclusions:**

Adolescent populations are particularly vulnerable to HIV, and oral PrEP in these populations is likely to have an impact on population-level HIV incidence. The challenges of disseminating an HIV biomedical prevention tool requiring daily usage in adolescents are formidable, but addressing these issues and starting dialogues will lay the groundwork for the many other HIV prevention tools now being developed and tested.

## Introduction

### Overview of adolescent HIV epidemic

According to the World Health Organization (WHO) [1], AIDS is the leading cause of death among adolescents in sub-Saharan Africa and second leading cause for adolescents worldwide. In 2014, there were an estimated 2 million adolescents aged 10 to 19 living with HIV, and in 2013, adolescents aged 15 to 19 were infected with HIV every 2 minutes [2]. A recent report by the United Nations Children's Fund [3] concludes that by the year 2050, the number of Africans under the age of 18 may swell to one billion. Thus, the success of ending the AIDS epidemic depends upon reaching adolescents and engaging them in HIV prevention.

Adolescents are at increased risk for HIV due in part to the multiple co-occurring transitions (i.e. biological and psychological) and developmental tasks (e.g. establishing identity) in this period of the lifespan [4–6], in addition to age and power imbalances [7,8], gender inequality, and interpersonal violence in sexual relationships [9–12]. Among youth, there are also subgroups who bear disproportionate burdens of HIV and are the most vulnerable. These young key populations include men who have sex with men (MSM), transgender people, those who inject drugs, male and female sex workers, as well as youth who belong in multiple groups (e.g. transgender youth who inject drugs). Finally, a compelling case has been made for considering adolescent girls in sub-Saharan Africa, a key population that urgently requires attention and intervention; girls aged 15 to 19 in this region are four to five times more likely to be infected than their male counterparts [13] and HIV incidence rates are 5 to 6% among young women <21 years in recent HIV prevention trials [14,15].

### Definition of adolescence

Both the WHO [16] and the United Nations identify adolescence as the period in human growth and development that occurs after childhood and before adulthood, from ages 10 to 19. For the purposes of this article, we consider adolescents those under the age of majority (i.e. the age at which a child becomes a legal adult) for the country (South Africa) or state (within the United States) that they live in. Although there are developmental similarities between those under the age of majority and those just over the age of majority, there are distinct implementation challenges for under age youth that we focus on in this article.

## Discussion

### Studies of PrEP among adolescents in the United States and South Africa

At the time of this writing, there are no data yet available from PrEP studies among adolescents. There are only two oral PrEP studies currently in the field that exclusively focus on adolescents, which we describe below, as well as several other adolescent-inclusive demonstration projects that began in 2016. Successful enrolment of adolescents into biomedical HIV prevention trials or bridging studies is critical for drug licensure and implementation of PrEP among populations most vulnerable to HIV.

#### ATN 113 (Clinical trial NCT01769456) – United States

The first adolescent PrEP study, funded through the Adolescent Medicine Trials Network (ATN) for HIV/AIDS Interventions, is ATN 113 (Project PrEPare). ATN 113 is a demonstration project and phase II safety study that aims to obtain additional data on the safety of TDF/FTC (Truvada^®^) and to evaluate patterns of use, rates of adherence, and patterns of sexual risk behaviour among young MSM aged 15 to 17. Multiple recruitment methods were employed across sites, including street and venue-based outreach, community and school presentations, and online advertising on social media websites and social networking apps. Of the 1873 individuals approached, 59% were ineligible due to lack of recent sexual activity, and 8% were eligible but declined participation. Enrolling an adolescent cohort of MSM into a PrEP trial required more time and alternative strategies than the young adult cohort enrolled in the parallel study of 18 to 22-year olds (ATN 110) [17]. Internet-based recruitment strategies were more likely than in-person strategies to find eligible adolescent participants. For this study, adolescents were permitted to provide self-consent. Because of this approach to consent, only half of the eligible study sites were allowed by their institutional review boards to participate in the study [18].

Seventy-eight young MSM (mean age=16 years) were enrolled into ATN 113 from six study sites in the United States (Boston, Chicago, Los Angeles, Memphis, New Orleans, and Philadelphia). The participants are racially/ethnically diverse, with 45% identifying as Latino/Hispanic and 35% as Black/African-American. Study participants engaged in an evidence-based behavioural risk reduction intervention prior to PrEP initiation were then seen monthly for the first 12 weeks of the study, then quarterly thereafter until 48 weeks. All participants are provided with sexual health promotion and adherence counselling at every visit. Study participation has been completed for the primary 48 weeks of the study, and data will be available soon [19]. An extension phase for those 56% of participants who met conservative, prespecified, renal, or bone criteria at the completion of study drug use is ongoing for an additional 48 weeks through October 2016.

#### CHAMPS PillsPlus (Clinical trials NCT02213328) – South Africa

The second study, CHAMPS PillsPlus, is an ongoing open-label study examining the safety, feasibility, and acceptability of daily oral Truvada as PrEP in HIV-negative adolescents. Ninety-eight female and 50 male participants aged 15 to 19 were recruited in two South African peri-urban settings in Johannesburg and Cape Town. Eligible female participants willing to use hormonal contraception and eligible male participants were consented for daily oral PrEP as part of a combination HIV prevention package that included condoms and sexually transmitted infection screening and treatment. Youth under the age of 18 were required to have parental/guardian consent for study participation. With a target enrolment of 150 youth, this study has completed enrolment and has easily enrolled adolescents (even with adult permission required and a six-month enrolment window) with a median age of 18 (IQR 17–19), more than half having a sexually transmitted infection at baseline and reporting condomless sex at some time (Linda-Gail Bekker, personal communication). Adolescents have been ineligible due to undiagnosed HIV positivity, pregnancy, or not yet sexually active by self-report. Initial plasma tenofovir levels indicate reasonable uptake and use of PrEP [20]. Recognizing that many youth struggle to consistently adhere to daily medication, this programme is also providing specific tailored support using SMS, adherence clubs, and real-time feedback on drug levels. Youth are also counselled by trained youth-friendly counsellors on the continuing need for PrEP with an option to opt out if risk profile has changed.

#### Other adolescent-inclusive studies

In addition to the above, a pilot study in Kenya is currently under way to examine the uptake, acceptability, and feasibility of a combination prevention package of gender-specific interventions for youth aged 15 to 24 in mobile health settings [21]. The prevention package for all youth includes HIV counselling and testing along with linkage to care for HIV treatment. PrEP is a component of the prevention package for young women who are out of school and test HIV-negative. However, preliminary results indicate that only 9% of those enrolled thus far may be eligible for PrEP, highlighting the need to learn how to target PrEP and reach higher risk young women [21].

Several new studies and demonstration projects that are inclusive of adolescents are set to begin this year. There are two new studies funded by the HIV research networks of the National Institutes of Health (NIH). The first is HIV Prevention Trials Network (HPTN) 082, a randomized multisite prospective study to assess PrEP acceptance and adherence among HIV-negative young women in South Africa and Zimbabwe, which has been launched this year (2016) among adolescent and young adult women, aged 16 to 25. In this study, young women who accept open-label daily oral PrEP were randomized to receive enhanced adherence counselling based on feedback from observed drug levels obtained in the first two months after PrEP initiation or standard adherence support. This study also follows women who initially decline PrEP and conducts qualitative research about factors that influence PrEP initiation and adherence. The second study is International Maternal Paediatric Adolescent AIDS Clinical Trials (IMPAACT) 2009, which is a parallel, observational cohort study designed to determine the feasibility, acceptability, and safety of oral PrEP among HIV-negative adolescents and young women during pregnancy and early breastfeeding. Participants for this study were enrolled in South Africa, Zimbabwe, Malawi, and Uganda.

Three additional adolescent-inclusive projects are slated to begin this year (2016). The first, a collaborative grant from the NIH and the South African Medical Research Council, will involve a prospective cohort evaluation of PrEP initiation and adherence among HIV-negative young women in Cape Town. This study will evaluate PrEP demand in the context of promotion and communication messages derived through formative research with young women in a Cape Town township and with social marketing about PrEP developed in collaboration with a creative marketing agency. This study will enumerate the proportion and characteristics of PrEP uptake among young women who are offered PrEP and will evaluate the effect of a modest incentive that is conditioned on study drug adherence in the first three months of PrEP use on subsequent PrEP adherence. The second study, POWER, which is funded by the US Agency for International Development (USAID), will involve PrEP delivery to young women in family clinics and youth clinics, and mobile testing programmes in Kisumu, Kenya, and Johannesburg and Cape Town, South Africa. End-user perspectives, scalable delivery strategies, PrEP adherence support interventions for young women, and cost-effectiveness will be evaluated in the context of PrEP delivery in a non-research setting. Finally, the third study, EMPOWER, which is funded by the United Kingdom Department for International Development, will involve PrEP delivery as part of a combination HIV prevention package that includes intervention to prevent gender-based violence and stigma. The study will evaluate the feasibility and acceptability of incorporating screening for gender-based violence into HIV counselling and testing and strategies for linkage to care, as well as offer PrEP to HIV-negative participants. The package of adherence support will include counselling and SMS reminders. Participants will be randomized to either adherence clubs that include a four-session empowerment curriculum focused on improving communication with sexual partners and addressing PrEP stigma or no clubs. PrEP adherence and retention in care at 12 months are the primary outcomes of interest.

### Regulatory approvals and clinical guidance for adolescents

As described elsewhere in this special issue, Truvada was approved by the US Food and Drug Administration (FDA) as PrEP in July 2012. The indication is for adults who are at high risk of contracting sexually acquired HIV. In November 2015, South Africa's Medicines Control Council approved PrEP for use among adults. Although the number of countries with PrEP approval is accruing rapidly (see map courtesy of AVAC), no country has approved a prevention indication for use with adolescents.

**Figure:**
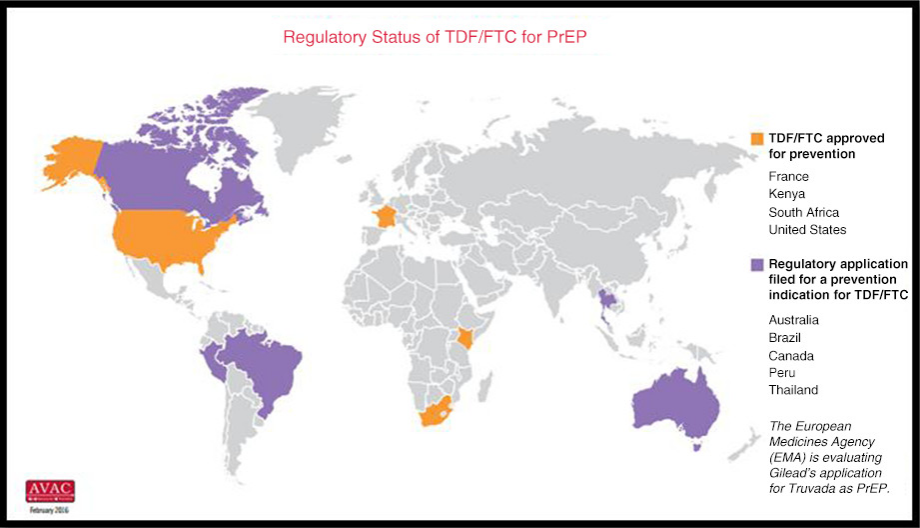


Despite regulatory limitations by age, several international groups have distributed recommendations that include considerations for adolescents. The US Centers for Disease Control and Prevention (CDC) released clinical guidelines in May 2014 [22] which state “currently the data on the efficacy and safety of PrEP for adolescents are insufficient. Therefore, the risks and benefits of PrEP for adolescents should be weighed carefully in the context of local laws and regulations about autonomy in health care decision-making by minors” (p. 9). However, some substantially affected metropolitan areas in the United States, such as New York City, have recently adapted slightly more permissive guidance (i.e. recommends PrEP be offered to adolescents at high risk for HIV infection) with appropriate caveats regarding lack of data and need for clinicians to consult institutional policies regarding parental consent [23].

In September 2015, WHO updated their recommendations on the use of PrEP to reflect that people at “substantial” risk of HIV should be offered PrEP with priority for populations with an HIV incidence of about 3 per 100 person-years or higher [24]. The WHO recommendations are not age-specific, but rather exclusively focused on risk level. Building upon these recommendations, WHO intends to release an implementation guide for PrEP this year (2016), which will also include adolescent-specific considerations. In preparation for the updated WHO recommendations and implementation guidelines, UNICEF organized an expert consultation on the implementation of oral PrEP in sexually active older adolescents aged 15 to 19 in July 2015. The report from that meeting outlines many key considerations for PrEP service delivery as well as identifying research gaps and advocacy needs [25]. Furthermore, although considerations for the cost-effectiveness of PrEP among adolescents and estimations of the population in need of PrEP have been presented in Table 1, [26], similar data for the United States have not yet been generated. However, research among young MSM, the population with the greatest HIV burden in the United States, consistently demonstrates incidence rates well above the 3% threshold suggested by WHO [27].

**Table 1:** Estimated number of people in need of PrEP in selected countries

Number of HIV-negative people and people living in sub-national areas with incidence ≥3%		Number of HIV-negative people and people living in sub-national areas with incidence ≥1.5%	

Characteristics	Estimates	Characteristics	Estimates
Women: • 15–19 years old • 20–24 years old	140,000 770,000	Women: • 15–19 years old • 20–24 years old	2.2 million 2.4 million
Men: • 15–19 years old • 20–24 years old	0 40,000	Men: • 15–19 years old • 20–24 years old	0 1.2 million
Countries: Kenya, Lesotho, Mozambique, South Africa, Swaziland.		Countries: Kenya, Lesotho, Mozambique, South Africa, Swaziland, Zimbabwe and Uganda.	

From Godfrey-Faussett *et al*. [26].

Thus, even in the absence of regulatory approval for the use of PrEP in adolescents, international guidelines have recognized that adolescents are a key population that is disproportionately impacted by the HIV epidemic worldwide. Global consensus on the need to focus PrEP implementation efforts on youth is very valuable for politicians and providers as they advocate for service delivery to youth. However, the impact of such guidance may be limited because, in the absence of regulatory approval, substantial structural, legal/ethical, and financial barriers continue to exist which effectively reduce access to youth populations most vulnerable to HIV. It is hoped that bridging studies, such as those described previously, will contribute the important safety and usability data needed to inform regulatory labelling of PrEP for younger populations.

### Implementation challenges

As the first country to approve PrEP in 2012, the United States has had the most experience to date with the challenges of PrEP implementation. In fact, recently presented data on PrEP prescriptions in the United States demonstrate that initiation of PrEP has been very low among youth – only 7.6% of all prescriptions were for patients under the age of 25 [28]. Thus, we present some of the barriers encountered by youth in the United States, followed by discussion of the potential barriers that may arise in South Africa as they consider national implementation of PrEP.

### PrEP implementation for adolescents in the United States

#### Clinician knowledge and comfort

Almost four years post-FDA approval of Truvada for PrEP in the United States, knowledge of PrEP and comfort with prescribing and monitoring the PrEP regimen remain limited among clinicians [29,30]. However, the providers thought to be likely to prescribe PrEP to adults (e.g. infectious disease or primary care physicians) may be very different than those who work directly with adolescents (e.g. paediatricians/adolescent medicine and family planning clinicians). A small, qualitative study of US-based clinicians specializing in adolescent medicine found that the CDC Interim Guidance (2012) on PrEP use was largely compatible with their practice [31]. However, there was still variability in clinician-reported characteristics of an appropriate PrEP patient based on assumptions about adherence, sexual risk, mental health, and substance-use issues. Some clinicians in this study voiced concerns about ethical/legal and cost barriers for youth under the age of 18, which made some report a preference for prescribing to those over 18 [31]. More recently, a survey of family planning clinicians found very low levels of PrEP knowledge. In fact, only 36% of respondents in this study had seen the PrEP clinical guidelines despite the importance of HIV prevention as a core service for family planning clinics [32]. Given that adolescents are probably less likely than adults to request PrEP at their primary care visits, the limited knowledge and remaining inadequacies in PrEP training among adolescent-focused specialties must be addressed comprehensively.

#### Off-label use and cost

Even if a surge in provider knowledge brought the option of PrEP to the forefront of adolescent care, the lack of FDA approval for those below age 18 in the United States creates barriers for youth, particularly because of the significant cost of the medication. For most adults who meet the indication for PrEP, the option of paying out of pocket for Truvada is cost prohibitive. In response to this, Gilead Sciences offers several programmes to assist with medication costs, including a medication assistance programme for those uninsured or underinsured as well as a co-pay assistance programme for insured patients with high co-pays. Because Truvada is only approved for use with “adults,” patients under the age of 18 are not eligible for these programmes. Furthermore, although most insurance companies will likely agree to pay for PrEP off-label eventually, it may take more benefit navigation and advocacy than most adolescents are prepared to tackle. Finally, although costs for the medication itself far exceed the costs of medical monitoring for PrEP, office visits, STI testing, and other laboratory work can create financial barriers that are unfamiliar to adolescents. Thus, adolescents at risk for HIV are even more financially vulnerable than adults and less likely to have unfettered access to important prevention tools.

#### Ethical and legal issues for adolescents

In the United States, local state laws vary tremendously in their language, or absence of language, about the rights of adolescents to access preventative services. Requirements for parental/guardian consent can greatly inhibit access and uptake of PrEP because requesting consent often forces unwanted disclosure of sexual activity and sexual orientation. Preliminary data from qualitative follow-up interviews of ATN 110/113 participants support these disclosure-related barriers [33]. There have been a few other studies documenting the variability in the legal and ethical interpretation of an adolescent's ability to seek PrEP with or without parental/guardian consent [18,34,35]. Although several of these studies examine the context of consent within the framework of the ATN 113 study, other literature has compared PrEP to family planning and other HIV prevention programmes [35,36]. Clarity in the laws is critically needed, with PrEP-specific language included whenever possible. However, even if adolescents are widely allowed to consent for themselves in the future, the cost issues described previously will still create barriers because parents will be notified of their child's PrEP use through insurance statements and explanations of benefits. Again, such unwanted disclosure is a substantial barrier that may result in avoidance of PrEP. Ultimately, this has significant policy-level implications for state regulations that need to be urgently addressed in parallel to the challenges already presented. One example of such progressive policies is the recent legislation introduced by the governor of New York which clearly states that adolescents under the age of 18 should be able to access both PrEP and HIV treatment without parent or guardian consent [37].

#### Youth-focused service delivery

CDC Clinical Guidelines [22] recommend quarterly visits for HIV testing and medical monitoring of PrEP. For adults who are used to seeing a physician one to two times per year, this may be seen as a burden and many care systems and insurance companies are trying to find ways to implement PrEP with minimal time and cost burden for patients. Unfortunately, this may not meet the needs for young people. In the qualitative study by Mullins and colleagues [31], adolescent providers expressed concern that the recommended visit frequency for PrEP monitoring was inadequate based on their expertise in caring for young people. The data from the ATN 110 study with 18 to 22-year olds appear to support this concern. Study participants were seen monthly for the first 12 weeks, then quarterly thereafter until 48 weeks. Results show levels of Truvada that can prevent HIV infection (i.e. ≥4 pills/week) were present for most participants during the monthly visits, but then dropped noticeably when the quarterly visit schedule began [17]. Preliminary data from ATN 113 show a similar, but even more striking drop in adherence with quarterly visits [19], and the CHAMPS PlusPills study is following a similar trend (Linda-Gail Bekker, personal communication). Thus, an augmented visit schedule may be preferable for young people, at least during PrEP initiation, with an even greater focus on adherence interventions especially during times of pill fatigue. Text messaging and mobile application interventions to improve PrEP adherence are currently being tested [38], which may be relatively simple ways to enhance adherence using technology that is highly familiar to youth. The use of adherence clubs and other social support groups has shown promise as a cost-effective strategy for HIV treatment adherence among adolescents and may be a strategy for PrEP users as well [39,40]. Although more comprehensive service provisions may increase cost, the resulting HIV prevention possibilities for youth will have broad social and economic benefits [41].

### Preparing for PrEP implementation among youth in South Africa

The South African government has prioritized the key populations of sex workers, MSM, and young women and girls for enhanced prevention programmes, including oral PrEP, and national guidelines are currently being written for the safe use of PrEP in each of these populations. In fact, the programme focused on sex workers was officially launched in June 2016. Initial plans in South Africa are to utilize the vertical service platforms that already exist in country for MSM and sex workers; however, the highly stigmatized nature of work with these groups does raise additional feasibility concerns. Notably, the South African Medicine Control Council has licensed the PrEP indication only for adult use at this time, leaving uncertainty regarding how to implement PrEP for adolescent minors. Questions have already been raised about which platforms can be safely and successfully used to provide PrEP to adolescents and how young women will be appropriately identified without stigmatization and labelling. Additional concerns exist about whether these programmes can be integrated into primary healthcare, including sexual and reproductive health services, antenatal care, or other services where youth can be reached. HPTN 067/ADAPT has provided some encouraging data about the willingness and feasibility of young women in South Africa to take daily PrEP. This study, which investigated daily versus intermittent PrEP, confirmed that better adherence and coverage of sexual events occurred with daily PrEP compared to intermittent dosing [42].

New demonstration projects have been encouraged and are being planned (e.g. Project SOAR and the DREAMS Initiative) as a way to better understand the implementation challenges for South Africa and other heavily impacted African countries. In addition, because a PrEP programme for young women will need to be much larger than one for young MSM or sex workers, there has been a great deal of concern raised about cost and sustainability, although strong evidence exists for the cost-effectiveness of PrEP among adults [43,44]. However, the evidence is strong for PrEP efficacy when adherence is high, and given ongoing high HIV incidence among young persons, there is a critical need to identify effective, scalable, and cost-effective PrEP delivery strategies for this key population.

## Conclusions

In conclusion, more scientific, programmatic, and policy work is needed to ensure that the adolescents that are most vulnerable to HIV infection will have appropriate access to PrEP. There are barriers to both PrEP access and uptake. To address access barriers, we must rectify the limited availability of youth-friendly health services in many settings, grapple with insurance and consent issues, including the implementation of consent policies and procedures appropriate for mature minors that avoid further marginalizing these at-risk youth from accessing efficacious prevention services as well as advocate for regulatory approvals that will inform these policies and allow PrEP access for adolescents. In parallel, social marketing and social media to reach adolescents and help them recognize their HIV risk and be motivated to use new prevention tools like PrEP will help to increase uptake. We also need to find and scale-up effective strategies to help support adolescents in decision-making about PrEP as well as adherence support while on PrEP. Adolescents, in particular, are likely to need enhanced, developmentally appropriate, and culturally tailored support for risk recognition and consistent use of a daily pill. Ongoing research and careful evaluation of a number of interventions to optimize identification of appropriate PrEP users as well as facilitators and enablers of consistent and effective use are urgently needed.

## References

[1] WHO (2014). Health for the world's adolescents: a second chance in the second decade.

[2] United Nations Children's Fund and the Joint United Nations Programme on HIV/AIDS (2015). All in to #EndAdolescentAIDS: launch document [Internet].

[3] UNICEF (2014). Generation 2030. Africa [Internet].

[4] Geier CF (2013). Adolescent cognitive control and reward processing: implications for risk taking and substance use. Horm Behav.

[5] Smith AR, Chein J, Steinberg L (2013). Impact of socio-emotional context, brain development, and pubertal maturation on adolescent risk-taking. Horm Behav.

[6] Romer D (2010). Adolescent risk taking, impulsivity, and brain development: implications for prevention. Dev Psychobiol.

[7] Hawkins K, Price N, Mussa F (2009). Milking the cow: young women's construction of identity and risk in age-disparate transactional sexual relationships in Maputo, Mozambique. Global Public Health.

[8] Hallett TB, Gregson S, Lewis JJ, Lopman BA, Garnett GP (2007). Behaviour change in generalised HIV epidemics: impact of reducing cross-generational sex and delaying age at sexual debut. Sex Transm Infect.

[9] Lane T, Osmand T, Marr A, Shade SB, Dunkle K, Sandfort T (2014). The Mpumalanga Men's Study (MPMS): results of a baseline biological and behavioral HIV surveillance survey in two MSM communities in South Africa. PLoS One.

[10] Kubicek K, McNeeley M, Collins S (2015). “Same-sex relationship in a straight world” individual and societal influences on power and control in young men's relationships. J Interpers Violence.

[11] Jewkes RK, Dunkle K, Nduna M, Shai N (2010). Intimate partner violence, relationship power inequity, and incidence of HIV infection in young women in South Africa: a cohort study. Lancet.

[12] Campbell JC, Baty ML, Ghandour RM, Stockman JK, Francisco L, Wagman J (2008). The intersection of intimate partner violence against women and HIV/AIDS: a review. Int J Inj Contr Saf Promot.

[13] Dellar RC, Dlamini S, Karim QA (2015). Adolescent girls and young women: key populations for HIV epidemic control. J Int AIDS Soc.

[14] Baeten JM, Palanee-Phillips T, Brown ER, Schwartz K, Soto-Torres LE, Govender V (2016). Use of a vaginal ring containing Dapivirine for HIV-1 prevention in women. N Engl J Med.

[15] Marrazzo JM, Ramjee G, Richardson BA, Gomez K, Mgodi N, Nair G (2015). Tenofovir-based pre-exposure prophylaxis for HIV infection among African women. N Engl J Med.

[16] World Health Organization (2016). Maternal, newborn, child and adolescent health: adolescent development [Internet].

[17] Hosek S, Rudy B, Landovitz R, Kapogiannis B, Siberry G, Liu N (2015). An HIV pre-exposure prophylaxis demonstration project and safety study for young men who have sex with men in the United States (ATN 110). J Int AIDS Soc.

[18] Gilbert AL, Knopf AS, Fortenberry JD, Kapogiannis BG, Hosek S, Zimet GD (2015). Adolescent self-consent for biomedical HIV prevention research: implications for protocol approval and implementation. J Adolesc Health.

[19] Hosek S, Landovitz R, Rudy B, Kapogiannis B, Siberry G, Rutledge B The adolescent trials network for HIV/AIDS interventions (ATN). An HIV pre-exposure prophylaxis (PrEP) demonstration project and safety study for adolescent MSM ages 15–17 in the United States (ATN 113).

[20] Gill K, Marcus R, Dietrich J, Bennie T, Hosek S, Gray G An analysis of baseline and early data from the Plus Pills study: an open-label trial of pre-exposure prophylaxis for South African adolescents.

[21] Kurth A, Buttolph J, Inwani I, Agot K, Cleland CM, Cherutich P Gender specific combination HIV prevention: baseline results from the MP3 youth pilot study.

[22] Centers for Disease Control and Prevention (2014). Clinical guidelines for the use of pre-exposure prophylaxis to prevent HIV.

[23] New York State Department of Health (NYSDOH) (2015). Guidance for the use of pre-exposure prophylaxis (PrEP) to prevent HIV transmission – revised.

[24] WHO (2015). Guideline on when to start antiretroviral therapy and on pre-exposure prophylaxis for HIV.

[25] (2015). UNICEF consultation on clinical, ethical and operational considerations for the implementation of oral pre-exposure prophylaxis (PrEP) in sexually active older adolescents (15–19) at high risk of HIV infection: meeting report.

[26] Godfrey-Faussett P, Ghys PD, Stover J, Mahy M, Daher J, Sabin K Estimated population of sexually active adolescents (15–19) at high risk of HIV infection in need of PrEP and cost implications.

[27] Sullivan PS, Rosenberg ES, Sanchez TH, Kelley CF, Luisi N, Cooper HL (2015). Explaining racial disparities in HIV incidence in black and white men who have sex with men in Atlanta, GA: a prospective observational cohort study. Ann Epidemiol.

[28] Bush S, Magnuson D, Rawlings MK, Hawkins T, McCallister S, Mera Giler R Racial characteristics of FTC/TDF for pre-exposure prophylaxis (PrEP) users in the US.

[29] Mimiaga MJ, White JM, Krakower DS, Biello KB, Mayer KH (2014). Suboptimal awareness and comprehension of published pre-exposure prophylaxis efficacy results among physicians in Massachusetts. AIDS Care.

[30] Karris MY, Beekmann SE, Mehta SR, Anderson CM, Polgreen PM (2014). Are we prepped for pre-exposure prophylaxis (PrEP)? Provider opinions on the real-world use of PrEP in the United States and Canada. Clin Infect Dis.

[31] Mullins TL, Lally M, Zimet G, Kahn JA (2015). Clinician attitudes toward CDC interim pre-exposure prophylaxis (PrEP) guidance and operationalizing PrEP for adolescents. AIDS Patient Care STDs.

[32] Seidman D, Carlson K, Weber S, Witt J, Kelly PJ (2016). United States family planning providers’ knowledge of and attitudes towards pre-exposure prophylaxis for HIV prevention: a national survey. Contraception.

[33] Knopf A, Ott M, Liu N, Kapogiannis B, Zimet G, Fortenberry D Unexpected benefits and heightened disclosure risks: adolescents’ experiences in a PrEP trial.

[34] Moore QL, Paul ME, McGuire AL, Majumder MA (2016). Legal barriers to adolescent participation in research about HIV and other sexually transmitted infections. Am J Public Health.

[35] Culp L, Caucci L (2013). State adolescent consent laws and implications for HIV pre-exposure prophylaxis. Am J Prev Med.

[36] Burda JP (2015). PrEP and our youth: implications in law and policy. Columbia J Gend Law.

[37] New York State Government website; Press Office https://www.governor.ny.gov/news/governor-cuomo-announces-new-legislation-end-aids-epidemic-new-york-state.

[38] Haberer JE (2016). Current concepts for PrEP adherence in the PrEP revolution: from clinical trials to routine practice. Curr Opin HIV AIDS.

[39] Bango F, Ashmore J, Wilkinson L, Cutsem G, Cleary S (2016). Adherence clubs for long-term provision of anti-retroviral therapy: cost-effectiveness and access analysis from Khayelitsha, South Africa. Trop Med Int Health.

[40] Grimsrud A, Lesosky M, Kalombo C, Bekker LG, Myer L (2016). Implementation and operational research: community-based adherence clubs for the management of stable antiretroviral therapy patients in Cape Town, South Africa: a cohort study. J Acquir Immune Defic Syndr.

[41] Stover J, Rosen J, Kasedde S, Idele P, McClure C (2014). The impact and cost of the HIV/AIDS investment framework for adolescents. J Acquir Immune Defic Syndr.

[42] Bekker LG, Hughes J, Amico R, Roux S, Hendrix C, Anderson PL HPTN 067/ADAPT Cape Town: a comparison of daily and nondaily PrEP dosing in African women.

[43] Ross EL, Cinti SK, Hutton DW (2016). A cost-effective, clinically actionable strategy for targeting HIV pre-exposure prophylaxis to high-risk men who have sex with men. J Acquir Immune Defic Syndr.

[44] Jewell BL, Cremin I, Pickles M, Celum C, Baeten JM, Delany-Moretlwe S (2015). Estimating the cost-effectiveness of pre-exposure prophylaxis to reduce HIV-1 and HSV-2 incidence in HIV-serodiscordant couples in South Africa. PLoS One.

